# Primary hepatic Burkitt lymphoma in a child and review of literature

**DOI:** 10.1186/s12957-023-03052-3

**Published:** 2023-07-24

**Authors:** Zhenhui Huang, Yuanxing Hu, Shengye He, Jiamin Zeng, Xin Zhang, Cuihong Ji, Haiwu Lu, Ping Xue, Xiaofeng Jiang

**Affiliations:** 1 Department of Hepatobiliary Surgery, Second Affiliated Hospital of Guangzhou Medical University, Guangzhou, China; 2 Department of Pathology, Second Affiliated Hospital of Guangzhou Medical University, Guangzhou, China

**Keywords:** Primary hepatic non-Hodgkin lymphoma, Primary hepatic Burkitt lymphoma, Childhood, Liver tumor

## Abstract

**Background:**

Primary hepatic Burkitt lymphoma (PHBL) in children is an extremely rare hepatic malignancy with a dismal prognosis, unless it is detected and treated promptly.

**Case summary:**

An 11-year-old child with abdominal pain was admitted to our hospital. No notable abnormalities were found during his physical examination or laboratory workup, but the abdominal computed tomography and magnetic resonance imaging both indicated a malignant hepatic mass measuring 9.2 × 7.1 × 7.5 cm in size. His postoperative pathology revealed an unexpected primary hepatic Burkitt lymphoma following a laparoscopic liver lobectomy. He then received rituximab and intense multi-agent chemotherapy as treatment. Despite post-chemotherapy bone marrow suppression, the patient eventually made a full recovery and had a good overall state.

**Conclusion:**

In this study, we describe a rare case of pediatric primary hepatic Burkitt lymphoma and review the literature on clinical features, diagnosis, and treatment for primary hepatic Burkitt lymphoma in children. We stress that this diagnosis should be taken into account in the absence of other single hepatic lesions or primary tumors of hematological disorders, particularly when there is a normal AFP level.

## Introduction

Primary hepatic lymphoma (PHL) is a rare cancer that accounts for less than 10% of all focal hepatic lesion [1]. Among lymphoma, PHLs make up 0.4% of all extranodal lymphomas and 0.016% of all non-Hodgkin lymphomas [2]. The diffuse large B cell lymphoma (DLBCL) tissue type is frequently observed in the PHLs [3, 4]. However, Burkitt lymphoma, a kind of histological type of mature B cell non-Hodgkin’s lymphoma that originates from liver without extrahepatic primary lesions, is highly uncommon in children with just eight cases reported globally (Table 1) [5–12]. We describe a rare case of primary hepatic Burkitt lymphoma (PHBL) in children in this report and review the prior literature to offer an overview of its epidemiology, etiology, clinical presentation, imaging features, pathological features, therapy, and prognosis.

**Table 1:**
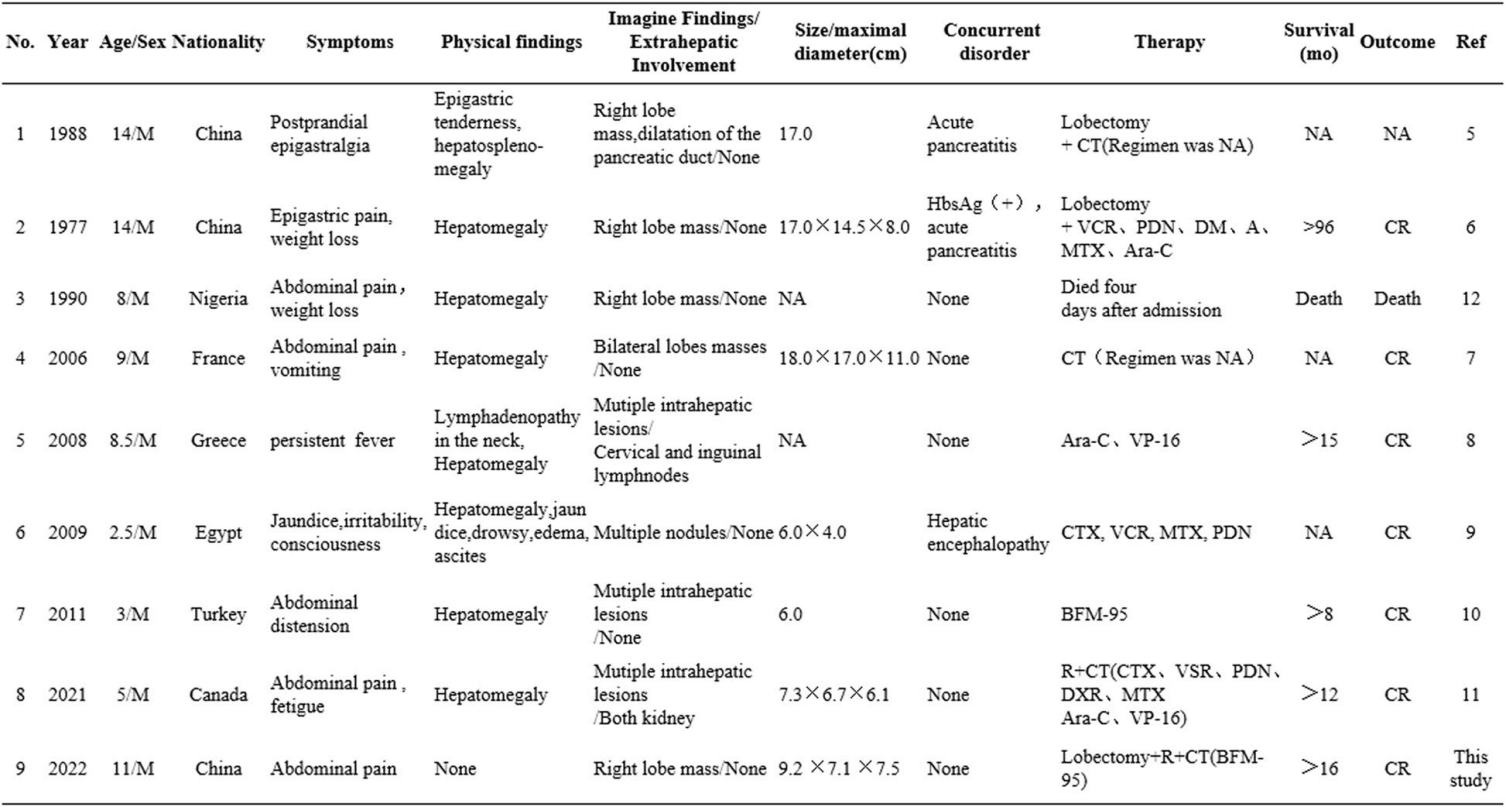
Clinical characteristics and treatment results of children with primary hepatic Burkitt lymphoma

*M* Man, *F* Female, *NA* not available, *Ara-C* Cytarabine, *VP-16* Etoposide, *CTX* Cyclophosphamide, *DXR* Doxorubicin, *MTX* Methotrexate, *PDN* Prednisone, *VCR* Vincristine, *R* Rituximab, *DM* Daunomycin, *A* Asparaginase, *CR* Complete remission, *CT* chemotherapy, *RT* Radiotherapy, *BFM* Berlin-Frankfurt-Munster protocol, *Ref* References

## Case presentation

An 11-year-old child who had been complaining of right upper quadrant ache for 10 days was hospitalized to our hospital. A large lesion that the local hospital discovered during a CT scan of his abdomen led them to believe that he most likely had liver cancer. He had no history of hypertension, coronary heart disease, diabetes, or infectious disease. His grandfather passed away from liver cirrhosis in the past, and his father carried the hepatitis B virus. Physical examination revealed that his belly was soft, flat, and lacking any abdominal mass or rebound pain.

**Fig. 1:**
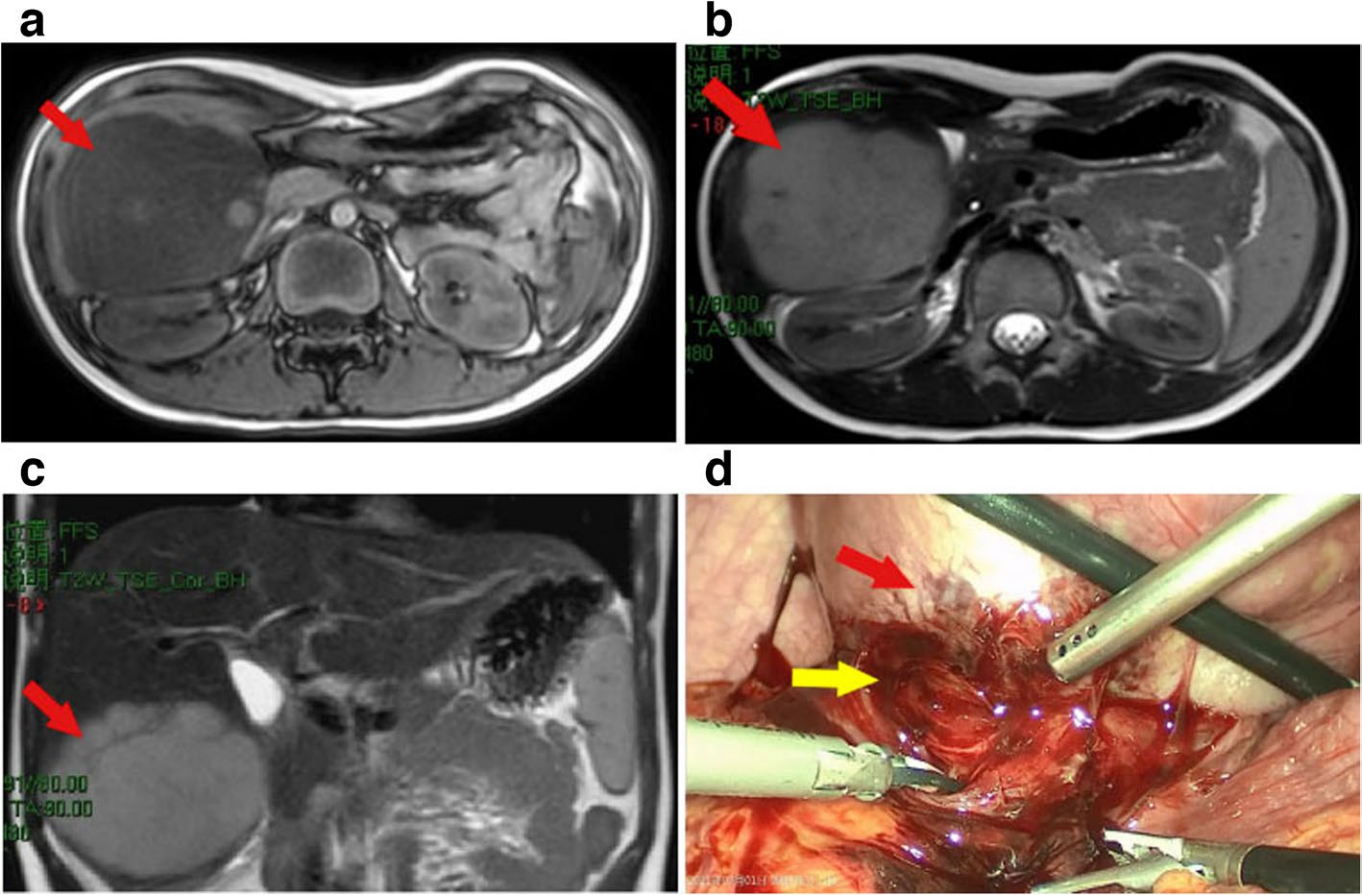
**A** T1w sequence, transverse plane, revealing a large space occupying lesion in the right lobe of liver. **B** T2w sequence, same plane, showing the hyper-intense signal of the lesion. **C** T1w sequence, coronal plane, following intravenous contrast administration. **D** Intraoperative picture. The tumor (red arrow) with spontaneous rupture and bleeding in the bottom was adhesion to the lower omental tissue (yellow arrow)

Laboratory workup for tumor markers, liver function, coagulation, blood routine, and liver function did not reveal any notable abnormalities. Our hospital’s abdomen enhanced CT scan revealed that the lesion, which measured 9.2 × 7.1 × 7.5 cm, was located in the hepatic segments V and VI. Additionally, it implied that the lesion might mostly be malignant. Moreover, hepatic MRI did not reveal any mesenteric or retroperitoneal lymphadenopathy, calcification, or necrosis (Fig. 1A–C). On the basis of this,we recommended him to receive the laparoscopic resection.

**Fig. 2:**
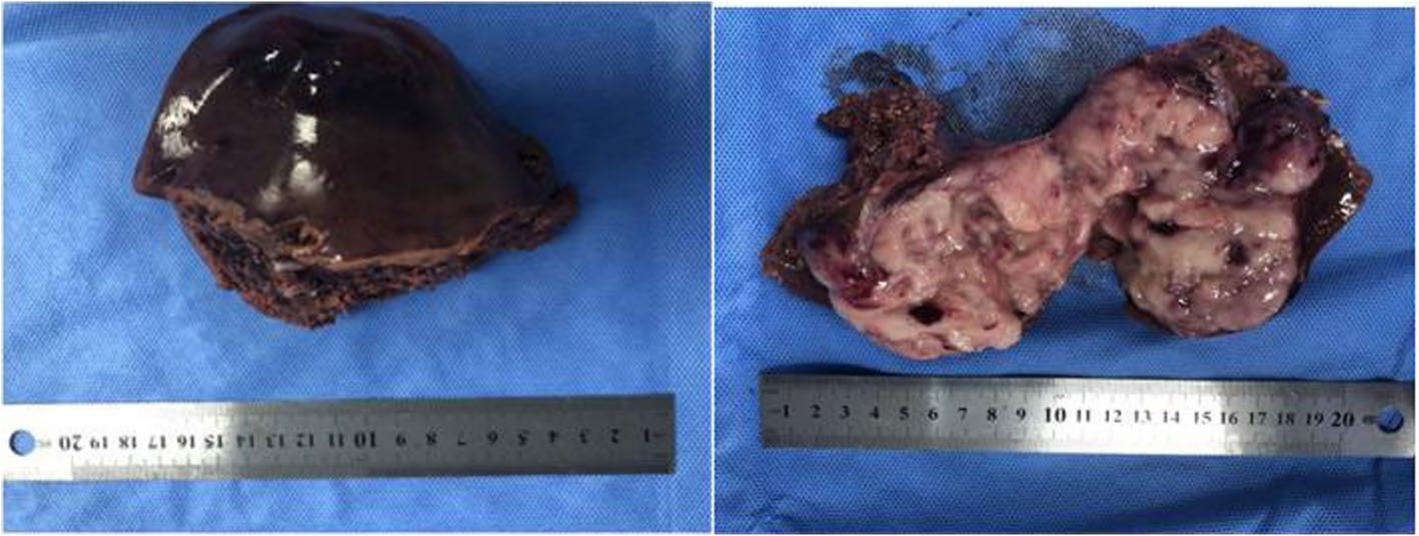
Massive grayish white necrotic tissue was found in the dissected specimen

During surgery, a massive solid mass with unclear boundaries was discovered protruding through the surface of the right lower lobe of the liver. Particularly, the tumor was adhering to the lower omental tissue with spontaneous rupture and bleeding under the tumor mass (Fig. 1D). No extra-hepatic metastases were seen, and no enlarged lymph nodes were also detected. Following removal of the mass from the abdominal cavity, a 9.2 × 7.1 × 7.5 cm tumor with localized bleeding and necrosis was discovered by pathological testing (Fig. 2). The specimen was then sent to pathology for diagnostic tests.

The surgery took 190 min, and 100 mL of blood was lost in total. After surgery, the patient made a full recovery without any blood transfusions. Unexpectedly, the definitive diagnosis of hepatic Burkitt lymphoma (PHBL) was made. This is an uncommon hematologic cancer of the liver. Consequently, after he was discharged from our hospital, we advised that he continue his treatment in a children’s specialty facility.

He received rituximab continuously along with chemotherapy in accordance with BFM Collaboration Group protocol. But frequent chemotherapy caused severe post-chemotherapy bone marrow suppression, which reduced the production of platelets, red blood cells, and white blood cells. He subsequently experienced recurrent fever and rash, although these symptoms were managed by symptomatic therapy such anti-infection, gamma globulin infusion, and blood transfusion. In the end, he successfully finished five rounds of chemotherapy within 4 months of the operation.

## Follow-up

We conducted a systematic follow-up on the patient 16 months following the operation. He made a good recovery and showed no signs of fever or redness. His laboratory tests also return to normal. And his PET-CT scan results indicate that there are no signs of recurrence, which is considered achieving complete remission (CR).

**Fig. 3:**
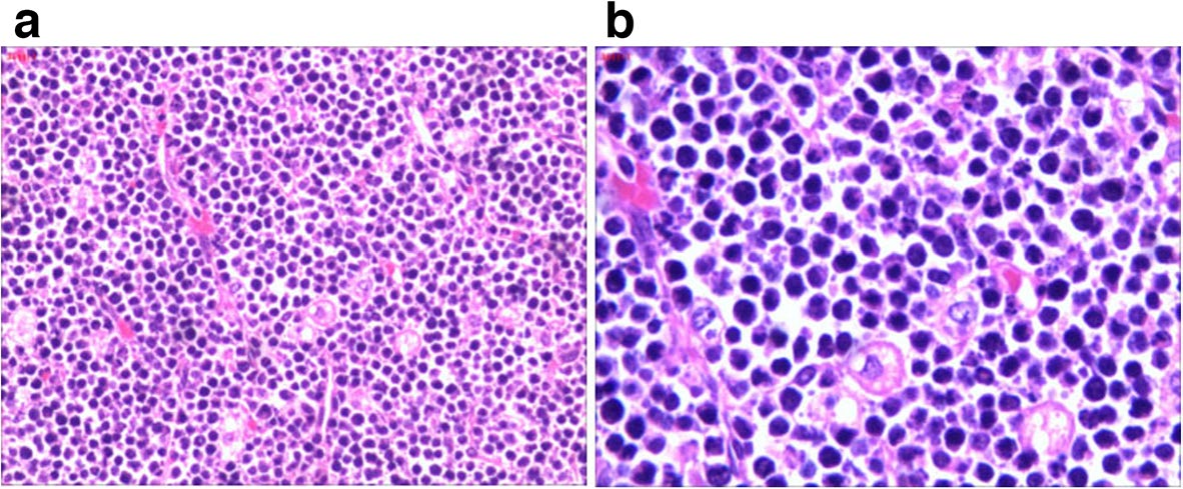
Hematoxylin and eosin stain (HE) of the tumor tissue. **a** HE, 100 × . **b** HE, 200 ×

## Discussion

Although secondary liver involvement in non-Hodgkin lymphomas is frequently observed in their advanced stages, primary hepatic lymphoma (PHL) is a rare type of cancer. According to previous study, man in their middle years are most frequently affected [13, 14].PHL constitutes 0.016% of all non-Hodgkin lymphomas [2]. As a kind of histological type of mature B cell non-Hodgkin’s lymphoma, Burkitt’s lymphoma (BL) is a highly aggressive and quickly developing cancer. Particularly, primary hepatic Burkitt lymphoma (PHBL), defined as BL confined to the liver is exceptionally rare at any age. According to our knowledge, only eight cases of PHBL in children have been reported in the literature that is currently available (Table 1).

Taking this present case into account, their median age was 8.4 years (range, 2.5–14 years). The clinical manifestations of PHBL are non-specific and include nausea, exhaustion, weight loss, and abdominal pain. The child only ever displayed clinical illness and symptoms of liver failure in one instance. Two cases in particular were complicated with acute pancreatitis and hepatectomy eased the symptoms. It was hypothesized that the extrinsic tumor compressing the ampulla of Vater was partially blocking the pancreatic outflow. Physical examination of PHBL may also exhibit no abnormalities. Nevertheless, common positive indications include hepatomegaly, with some instances also showing jaundice or lymphadenopathy. Corresponding with adult data [13, 14], all of the known pediatric PHBL cases are in boys, and none of them show signs of a pre-existing liver condition. In our research, the only documented fatality was that of a youngster whose diagnosis illness did not allow him to live long enough to begin therapy, and his diagnosis of PHBL was made post-mortem [11]. Nevertheless, chemotherapy alone or in combination with surgical resection worked successfully for the majority of kids.

Despite individuals with PHL being discovered to be related with a number of diseases, such as EBV, HBV, HCV, or HIV infections, liver cirrhosis, systemic lupus erythematosus, and immunosuppressive medication, the pathogenesis of PHL has not yet been thoroughly elucidated [13, 15]. Only one of the nine children in this study had HAV, and none of the others had any complications from the aforementioned illnesses. It is unfortunate that genetic screening was not done on the youngster in this circumstance. Accordingly, more pediatric case studies and analysis are required.

Owing to its rarity, PHBL may be difficult for doctors and pathologists to diagnose. Primary hepatic tumors and metastatic lesions are included in the initial differential diagnosis made only on the basis of a physical examination. It is important to distinguish primary hepatic tumor from hepatoblastoma, hepatocellular carcinoma, hepatic adenoma, and other related conditions. The diagnosis of hepatoblastoma and hepatocellular carcinoma in our patient was not supported by the normal AFP. Hepatic adenomas often appear during puberty and are linked to androgen use [10], which was not the case in our patient. Infantile hemangioendothelioma is unlikely because the disease typically manifests before the age of 6 months as a mass in the liver or with severe hepatomegaly and heart failure brought on by many arteriovenous connections inside the tumor. The biliary tract is where hepatic embryonal sarcoma develops, and it typically manifests before the age of 5. Since there are no associated symptoms, the typical presentation, which is jaundice secondary to biliary blockage, is improbable in this instance. In our situation, the possibility of a primary hepatic neuroendocrine tumor was strongly considered because it might manifest as metastatic disease without a primary tumor. Additionally, immunohistochemistry results that are positive for chromogranin A (CgA) and neuron-specific enolase (NSE) are required to support this verified diagnosis. With regard to metastatic lesions, we could quickly rule out liver metastasis from an intestine tumor using common tumor markers as CA 19–9 and CEA. Finally, the lack of a primary kidney lesion on imaging examinations allowed for the easy exclusion of a Wilms tumor.

**Fig. 4:**
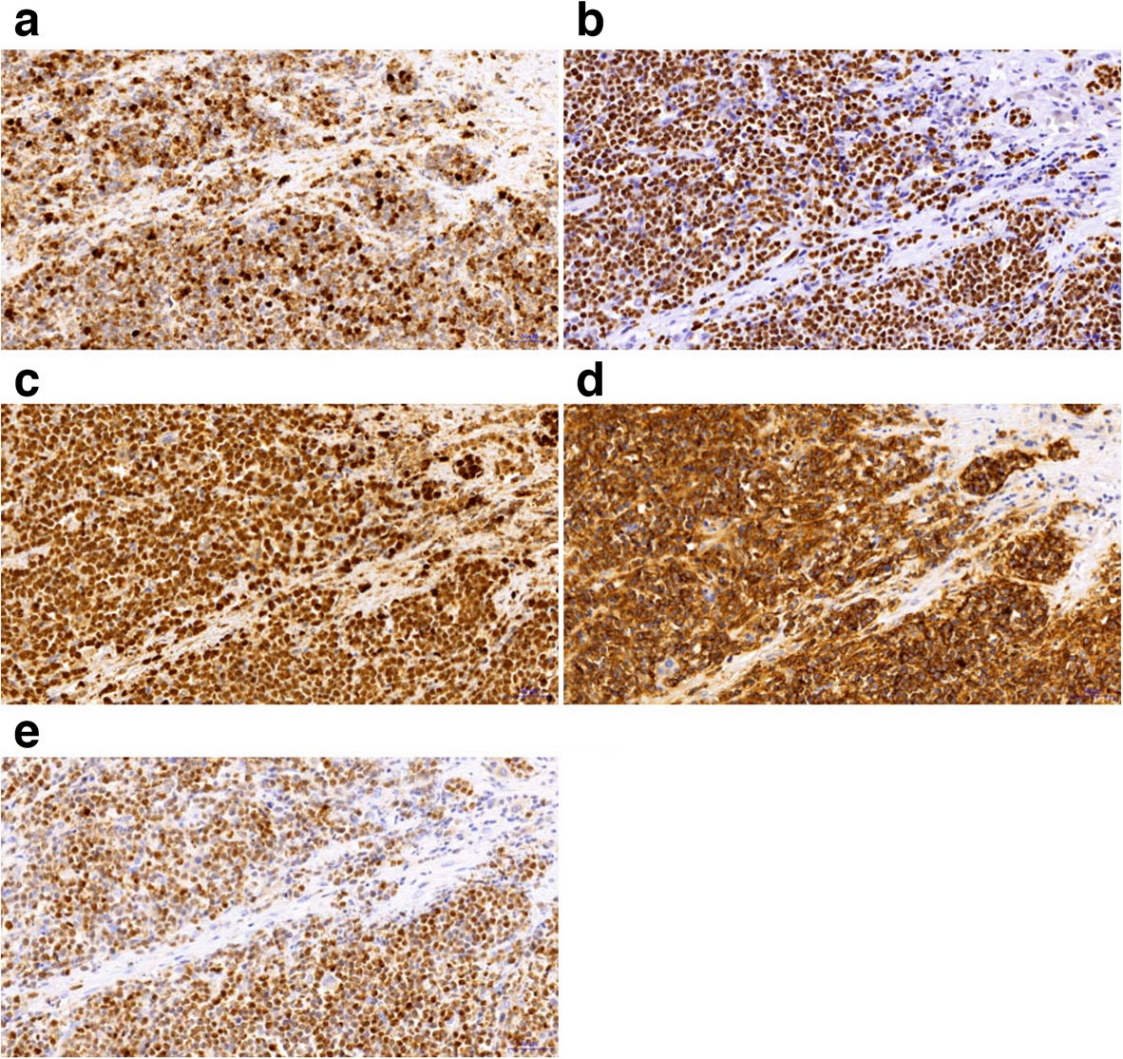
Immunohistochemical staining of the resected specimen. **a** Bcl-6( +), **b** CD20( +), **c** C-myc( +), **d** Ki67( +), **e** MUM1( +)

Histopathology provides the foundation for the PHL diagnosis in its entirety. It is acceptable to diagnose liver tumors using ultrasound-guided core-needle biopsy, but malignant tumors run the danger of metastasizing along the needle tract. Laparotomy also offers the additional advantage of allowing for a better examination of the abdominal cavity to rule out the presence of extra hepatic masses. Although the histological type of the mass was unclear, we performed a laparoscopic exploration and planned biopsy since it was possible that the tumor was malignant. Upon entering the abdominal cavity, we found that the liver tumor was huge, accompanied by the appearance of spontaneous rupture and bleeding (Fig. 1D), which was further suggestive of malignancy. Laparoscopic liver resection was consequently carried out directly. Massive grayish white necrotic tissue was found in the liver following the removal of the tumor, as seen in Fig. 2. The tumor cells were identified through additional pathological analysis as tiny to medium-sized cells with basophilic cytoplasm, fatty vacuoles, round nuclei, and condensed chromatin, together with admixed macrophages that displayed the recognizable “starry-sky” appearance (Fig. 3). Immunohistochemical staining revealed the abundant presence of the markers CD20, C-myc, Bcl-6, and MUM1. The proliferation factor determined by Ki67 was > 90%, which supported the Burkitt lymphoma diagnosis (Fig. 4).

According to Table 1, PHBL most frequently happens in the right lobe of the liver when it manifests as an isolated mass. Typically, ultrasound findings show the existence of hypoechoic homogeneous masses that correlate well with CT imaging data. However, the presentation of PHBL on MRI varies, and it may present as a single or multiple nodules. In general, hepatic lymphoma is typically described as hypointense to normal liver on T1-weighted images and hyperintense to liver on T2-weighted images [9, 16].

There is currently no agreed-upon treatment for this rare case, and reports of sole surgical therapy, sole chemotherapy, or combined therapy exist. According to recent research and our case, complete surgical resection would be the preferred choice of treatment for patients with a single tumor restricted to one side of the liver when the histologic diagnosis is unknown. According to Table 1, surgical resection combined with postoperative adjuvant chemotherapy seems to be a good choice for patients with good liver function for which reduction of tumor load by surgery may be an adjunct to chemotherapy to some extent. For individuals with numerous nodules or diffuse distribution of intrahepatic lesions, chemotherapy alone is preferable. Most pediatric oncology cooperative groups, as well as individual institutions, have reported excellent outcomes with intensive multiagent chemotherapy regimens that include cyclophosphamide, anti-metabolites, anthracyclines, etoposide, vincristine, and corticosteroids in the treatment of Burkitt’s lymphoma in children [4, 17, 18]. Additionally, it has been demonstrated that the level of serum lactic dehydrogenase (LDH) is suggestive to reflect the patient’s tumor load [4]. In other words, the increase of serum LDH may be a sensitive indicator of poor prognosis in PHBL.

## Conclusion

In conclusion, there is little knowledge about the causes and processes of pediatric PHBL, which is highly uncommon in kids and predominantly affects boys and kids in school. As a result, no established consensus treatment protocols exist at this time. It is also simple to make a mistaken diagnosis because PHBL’s clinical signs and laboratory tests are not specific, and the final diagnosis is still determined by pathology. Therefore, this diagnosis should be taken into account in the absence of other single hepatic lesions or primary tumors of hematological disorders, especially when the AFP level is normal.

## Data Availability

All data generated or analyzed during this study are included in the article. Further inquiries can be directed to the corresponding authors.

## Abbreviations

CT: Computed tomography
MRI: Magnetic resonance imaging
PHL: Primary hepatic lymphoma
PHBL: Primary hepatic Burkitt lymphoma
DLBCL: Diffuse large B cell lymphoma
EBV: Epstein-Barr virus
HBV: Hepatitis B virus
HCV: Hepatitis C virus
HIV: Human immunodeficiency virus
HAV: Hepatitis A virus
CEA: Carcinoembryonal antigen
AFP: Alpha-fetoprotein
LDH: Lactate dehydrogenase
CgA: Chromogranin A
NSE: Neuron-specific enolase
BFM: Berlin-Frankfurt-Münster protocol for B cell non-Hodgkin lymphoma

## References

[1] Sans M, Andreu V, Bordas JM (1998). Usefulness of laparoscopy with liver biopsy in the assessment of liver involvement at diagnosis of Hodgkin’s and non-Hodgkin’s lymphomas. Gastrointest Endosc.

[2] Noronha V, Shafi NQ, Obando JA, Kummar S (2005). Primary non-Hodgkin’s lymphoma of the liver. Crit Rev Oncol Hematol.

[3] Page RD, Romaguera JE, Osborne B (2001). Primary hepatic lymphoma: favorable outcome after combination chemotherapy. Cancer.

[4] Peng Y, Qing AC, Cai J, Yue C, French SW, Qing X (2016). Lymphoma of the liver: clinicopathological features of 19 patients. Exp Mol Pathol.

[5] Wan YL, Chen WJ, Huang SC, Lee TY, Tsai CC (1988). Solitary hepatic Burkitt lymphoma presenting as acute pancreatitis. Pediatr Radiol.

[6] Huang CB, Eng HL, Chuang JH, Cheng YF, Chen WJ (1997). Primary Burkitt’s lymphoma of the liver: report of a case with long-term survival after surgical resection and combination chemotherapy. J Pediatr Hematol Oncol.

[7] Fikri M, Dafiri R (2006). Primary hepatic lymphoma: report of a pediatric case. J Radiol.

[8] Mantadakis E, Raissaki M, Tzardi M, Katzilakis N, Chatzimichael A, Kalmanti M (2008). Primary hepatic Burkitt lymphoma. Pediatr Hematol Oncol.

[9] Al-Tonbary Y, Fouda A, El-Ashry R, Zalata K (2009). Primary hepatic non-Hodgkin lymphoma presenting as acute hepatitis in a 2-year-old male. Hematol Oncol Stem Cell Ther.

[10] Citak EC, Sari I, Demirci M, Karakus C, Sahin Y (2011). Primary hepatic Burkitt lymphoma in a child and review of literature. J Pediatr Hematol Oncol.

[11] Chamberlain G, Coltin H, Klaassen RJ, Story E, Abbott LS (2021). Successful treatment of pediatric primary hepatic Burkitt lymphoma using rituximab: a case report. Pediatr Blood Cancer.

[12] Adeodu OO, Nwosu SO, Odunusi OE (1990). Hepatic Burkitt lymphoma. Presentation with extremely rapid growth. Clin Pediatr (Phila).

[13] Ugurluer G, Miller RC, Li Y (2016). Primary hepatic lymphoma: a retrospective, multicenter rare cancer network study. Rare Tumors.

[14] Lei KI (1998). Primary non-Hodgkin’s lymphoma of the liver. Leuk Lymphoma.

[15] Dantas E, Santos J, Coelho M (2020). Primary hepatic lymphoma in a patient with cirrhosis: a case report. J Med Case Rep.

[16] Karadeniz C, Oguz A, Citak EC (2007). Clinical characteristics and treatment results of pediatric B-cell non-Hodgkin lymphoma patients in a single center. Pediatr Hematol Oncol.

[17] Patte C, Auperin A, Michon J (2001). The Société Française d’Oncologie Pédiatrique LMB89 protocol: highly effective multiagent chemotherapy tailored to the tumor burden and initial response in 561 unselected children with B-cell lymphomas and L3 leukemia. Blood.

[18] Spreafico F, Massimino M, Luksch R (2002). Intensive, very short-term chemotherapy for advanced Burkitt’s lymphoma in children. J Clin Oncol.

